# The Health Temples of Ancient Greece

**Published:** 1914-02-07

**Authors:** 


### THE HEALTH TEMPLES OF ANCIENT GREECE.

AT a recent meeting of the History of Medicine Section, Royal Society of Medicine, Sir Francis Champneys, Bart., in the chair, Professor Richard Caton delivered a very interesting lecture entitled "The Health Temples of Ancient Greece, and the Work carried on in Them."

He explained that much remained uncertain in regard to methods of treatment. Egypt seemed to have preceded Greece in the matter of medical lore and the accompanying medical superstition. It was difficult to say how far Greece learned from Egypt, but certainly many Egyptian methods were transported into Greece. The reference, in the Odyssey, Book IV., to the superiority of Egypt in medical matters was one proof of this, and the strange custom of incubation, long practised in Egypt, was another. In the dim twilight of Greek history Apollo was a health god. The chief later ones were Amynos, Amphiaraos, and Dexion. The occult Amynos had for centuries been forgotten; and there was no reference to him in Greek literature, except later in the writings of a Christian father. Amynos was the Athenian God of Healing, and the word meant to guard, help, and defend. Professor Dörpfeld laid bare the city which had been forgotten for eighteen centuries; he revealed the house and shrine of Amynion. The town consisted of small houses and narrow streets. It stood on the slopes of the Areopagus. During the excavations a curious irregularly-shaped area was disclosed, opening into a main street. The foundation-stones were of blue limestone, and the walls consisted of sun-dried brick, covered with hard cement, and the columns were rough pine trunks. Judging by the pottery found—the only guide—the date was between 900 B.C. and 1000 B.C., as the pottery was Proto-Corinthian and Greek Geometrical. A relief found in the ruins represented suppliants coming to the god for healing. Grateful patients were accustomed to presenting models, in gold or silver, of the limb or organ diseased, as an offering to the gods. One such was a huge leg, on which varicose veins were clearly represented. In 420 B.C. Æsculapius came to Athens, and his great temple on the east side was built. He was probably received in the sanctuary by Sophocles. Amynos was the pre-eminent god, and remained so up till Roman times.

### A VERY EARLY HOSPITAL WARD.

We did not hear much in this country about Amphiaraos. His shrine was built in a beautiful plain, clothed with trees and watered by a stream, in which numerous nightingales lifted up their voices. The temple was about 95 feet long and 43 feet wide, and had a broad column and portico. In front of the temple stood a large altar of limestone, 28 feet long and 14 feet broad, dedicated to Amphiaraos and several other gods. There were a number of long curved steps of white marble, on which, no doubt, the devotees of the sacrifice on the altar stood. There was also a sacred spring. North-east of the temple stood a building, 360 feet long by 36 feet broad. It was an open Doric colonnade, and there were forty-nine columns. Here was one of the earliest examples of a hospital ward, and it was the place in which incubation occurred. Between the nurses' kitchen and the ward was a grid, through which the patients could be watched. Down the centre of the ward ran a partition, women occupying the western side and men the eastern. Behind this long building stood a Greek theatre. One of the features of Greek medicine in all these great health temples was the need which was felt for keeping the patients amused. Professor Caton said he knew only one modern hospital which had amusements for the patients. In this Greek theatre was the great circular orchestra, the stage, and, scooped out of the hill-side, was a large auditorium for the sick. Further along was a bathing establishment, with ten large bath-rooms, separate rooms for men and women. It was founded in the fifth century B.C., and was open eight or nine months in the year—not in the winter. The priest was obliged to be present ten days in the month, and never absent more than three days together. Every patient paid a fee, and if he misbehaved himself he was fined a large amount. The priest was required to pray over the victims when sacrificed at the altar, and one or both shoulders of the sacrificed animal constituted the priest's fee; he also had a right to the skin. No part of the animal must be taken beyond the hospital. Before incubation every patient had to sacrifice a ram, and after he had eaten its flesh he lay night after night on its skin, hoping for dreams or visions, or even interviews with the god. It was not known whether drugs were given, but a special dietary, and particularly abstinence, was enjoined, which was probably beneficial after the repletion induced by eating the ram's flesh. No beans must be eaten. On leaving the hospital the patient was required to drop certain coins into the spring, and no doubt the priest sought a convenient opportunity to abstract them therefrom. All the health gods had an association with snakes, and pictures were shown of these animals being fed, golden models of limbs being in evidence.

The shrine of Trophonius, in Thebes, had a similar vogue for the treatment of sickness. A more important matter was that concerning the god Æsculapius, dating back to Homeric times. Epidaurus was the centre, from which it spread to all parts of Greece, and eventually to most of the Roman Empire. The site was six miles inland from the town of Epidaurus. It occupied a most beautiful and fertile valley. There was a magnificent theatre, accommodating 12,000 people; a huge hostel, and a beautiful grove. At the entrance to the establishment was a well, at which the suppliant had to undergo certain ceremonial ablutions. The front of the temple was of very fine Doric architecture, richly coloured. The door consisted of pure carved ivory, and in the various niches was exquisite statuary. Inside, at the western end, stood gigantic statues of Zeus and Athena. Pictures were shown of patients, who brought their own bed-clothes, lying at night awaiting dreams.

Another temple was the famous Thymenæ, built in the third century B.C. Beneath the foundations was a labyrinth, which some regarded as the sacred well; but it would not hold water, and there was no cement to lead to the view that it was intended to do so, nor any conduit. The lecturer felt, no doubt, that it was really the lair of the sacred serpents, where they dwelt and multiplied. The needed darkness was also secured. There were apertures in the floors, through which the snakes were brought, and patients fed them with honey cakes and other sacrificial delicacies.

The temple of Artemis Hecate was erected in the fifth century B.C. to one who was particularly kind to the young. It was the place of sacred fire, and there was a sacred spring. It contained a library, dedicated to Apollo and Æsculapius; and there was also a gymnasium, and a music-hall. A building, 90 yards square, contained eighty or ninety rooms, and was probably a hostel for the families of patients. A series of Roman baths were shown, constructed about the first century B.C.

In connection with the temple of Aphrodite was a great theatre, excavated out of the hill in the fourth century B.C., and it was said to be the most beautiful and interesting of the Greek theatres of the time. From the auditorium rose fifty-five rows of marble seats, and the acoustics of the building were wonderful: an even whispered recital at the stage could be heard at the top. There were some women who acted as priestesses at the hospitals; he did not know whether they officiated as nurses. There were impressive ceremonials, which must have had a good effect, especially in nerve cases: the sick man would lay his hand on the altar, and the priest would pray to the god to heal him. Then came the very important question of incubation. As the darkness fell, the lamps would be lighted, and the sick folk would bring their beds and recline on the couches. The priest then entered, and recited prayers to the gods. One, which had come down in the writings of Aristides, ran:

"Oh ye children of Apollo, who for so many of us in past time have stilled the waves of sorrow, and shown light and safety, be pleased to receive our prayer which will inspire in sleep and in vision. Order our worship aright, we pray you, according to your loving kindness; preserve us from sickness, give the body such measure of health and strength as may enable him to obey the spirit within, that our days may be passed in healthy vigour and in peace." That he regarded as a very beautiful prayer. The snake was the incarnation of the god. With darkness, silence was enjoined, and patients were urged to sleep and hope for visions. The serpents visited the sick during the night, and their visits were welcome, for they were tame and harmless, and they licked the sores. The inscriptions spoke of the god appearing to the patients during the night. A Greek was paralysed, and in his sleep he was told to go out on to the hill-side and bring in the largest stone he could find. He did so, and brought a huge stone, which was preserved as a memento of his cure. It would be very difficult for the ordinary man to lift it. Doubtless, it had a great moral effect on generations of future patients. A blind man had his sight restored at the hospital, and he returned home promising to send the fees on his arrival there. He did not send them, however, and the god made him blind again. He re-visited the hospital, paid his dues, and was permanently cured. Some of the practices showed a depth of superstition difficult for us to conceive. Later, science and art found a greater place in healing processes, for much attention was devoted to diet, plain hot and cold baths, oxide of iron, squills, lime-water, drugs to allay pain, gymnastic exercises, bleeding. The latter was portrayed in several reliefs shown on the screen.

But in every Greek shrine birth and death were absolutely prohibited. Sometimes a woman whose maternity was imminent was expelled, so that she was denied the attention most needed.

